# Midnolin Correlates With Anti‐Tumour Immunity and Promotes Liver Cancer Progression Through β‐Catenin

**DOI:** 10.1111/jcmm.70472

**Published:** 2025-03-20

**Authors:** Shaobo Huang, Jinling Zhang, Ting He, Jianping Zhou, Zhigang Liu

**Affiliations:** ^1^ Cancer Center, the Tenth Affiliated Hospital Southern Medical University (Dongguan People's Hospital) Dongguan China; ^2^ Dongguan Key Laboratory of Precision Diagnosis and Treatment for Tumors, the Tenth Affiliated Hospital Southern Medical University (Dongguan People's Hospital) Dongguan China; ^3^ Department of Medical Oncology Sun Yat‐Sen University Cancer Center, State Key Laboratory of Oncology in South China, Collaborative Innovation Center for Cancer Medicine, Sun Yat‐Sen University Guangzhou China; ^4^ School of Basic Medical Sciences Southern Medical University Guangzhou China; ^5^ Department of Thoracic and Cardiovascular Surgery The Tenth Affiliated Hospital, Southern Medical University (Dongguan People's Hospital) Dongguan China

**Keywords:** β‐Catenin, cancer, immune infiltration, MIDN, prognosis

## Abstract

Midnolin (MIDN) is a protein coding gene that promotes the destruction of transcription factors encoded by immediate‐early genes. Previous research has found that those immediate‐early genes are involved in tumour progression. However, the role of MIDN is still not clearly identified in human cancers. With the help of the TCGA, GTEx, and HPA databases, we revealed that the expression of MIDN was disordered in cancers. MIDN is a potential prognostic biomarker in liver cancer and bladder cancer. Prognostic analysis indicates that the expression level of MIDN gains survival benefits or promotes progression in multiple tumours. After analysing the sequencing results of TCGA via Gene Set Enrichment Analysis (GSEA), results suggested the regulative role of MIDN in cell proliferation and tumour immunity. Single cell sequencing results revealed that MIDN is highly expressed in several tumour tissues and also expressed in immune cells. With the help of the ESTIMATE, TIMER, and CIBERSORT databases, we analysed the immune score, immune cell infiltration, and anti‐cancer immunity cycle depending on the expression of MIDN. Results showed that low MIDN levels are tightly associated with high CD4 + T and NK cell infiltration. Furthermore, mutations of MIDN in cancers were significantly associated with immune cell infiltration. This study presents a robust link between the expression of MIDN and tumour progression across multiple cancer types. The MIDN/CTNNB1/MMP9 axis promotes liver cancer progression via inducing a suppressive tumour immune microenvironment.

**Abbreviations**
ACC: adrenocortical cancer
ANOVA: analysis of variance
ATCC: American Type Culture Collection
BLCA: bladder cancer
BRCA: breast cancer
CESC: cervical cancer
CHOL: bile duct cancer
CIBERSORT: cell‐type identification by estimating relative subsets of RNA transcripts
COAD: colon cancer
CTNNB1: catenin beta 1
DEG: Differentially Expressed Gene
DFS: disease‐free survival
DLBC: large b‐cell lymphoma
DMEM: Dulbecco's Modified Eagle Medium
DNA: deoxyribonucleic acid
DSS: disease‐specific survival
ECL: enhanced chemiluminescence
ESCA: esophageal cancer
ESTIMATE: estimation of stromal and immune cells in malignant tumor tissues using expression data
FBS: fetal bovine serum
FDR: False Discovery Rate
GBM: glioblastoma
GEPIA: Gene Expression Profiling Interactive Analysis.
GSEA: gene set enrichment analysis
GTEx: genotype‐tissue expression
HNSC: head and neck squamous cell carcinoma
HPA: human protein atlas
IFN: interferon
IL: interleukin
KICH: kidney chromophobe
KIRC: kidney clear cell carcinoma
KIRP: kidney papillary cell carcinoma
LAML: acute myeloid leukemia
LGG: lower grade glioma (LGG)
LIHC: liver cancer
LUAD: lung adenocarcinoma
LUSC: lung squamous cell carcinoma
MESO: mesothelioma
MIDN: midnolin
MSI: microsatellite instability
MTT: Methyl thiazolyl tetrazolium
NES: Normalized Enrichment Score
OS: overall survival
OV: ovarian cancer
PAAD: pancreatic cancer
PCPG: pheochromocytoma & paraganglioma
PFS: progression‐free survival
PRAD: prostate cancer
PVDF: polyvinylidene difluoride
READ: rectal cancer
SARC: sarcoma
sCNA: Somatic copy number alterations
SKCM: melanoma
SPF: Specific pathogen Free
STAD: stomach cancer
TCGA: the cancer genome atlas
TGCT: testicular cancer
TGFβ: transforming growth factor‐β
THCA: thyroid cancer
THYM: thymoma
TIMER: tumor immune estimation resource
TISCH: tumor immune single cell hub
TMB: tumor mutation burden
TME: tumor microenvironment
TNFα: tumor necrosis factor α
UCEC: endometrioid cancer
UCS: uterine carcinosarcoma
UV: Ultraviolet
UVM: ocular melanomas

## 1 Introduction

Global cancer statistics revealed that the incidence and mortality of cancer have climbed rapidly around the world [1, 2]. However, it is currently difficult to identify universal tumour biomarkers, so it is difficult to be diagnosed at an early stage [3]. In addition, different loci of tumours have tissue specificity, and the corresponding treatment is quite different [4]. Targeted therapy and immunotherapy have gained considerable clinical benefits, yet the high heterogeneity of tumours is still a challenge for precision medicine [5]. Significant genomic differences exist among cancers [6]. However, various cancer cells share some similar characteristics in growth [7]. The challenge currently faced in clinic is how to identify potential associations against a backdrop of differences. Usually, new functions of proteins are discovered in life science, and those functions are probably associated with diseases [8]. Those discoveries allow us to gain a deeper understanding of the differences and associations between various cancers. Therefore, exploring proteins with new functions in cancer is of great significance for tumour therapy and developing new therapeutic targets. The urgent need in current cancer treatment is to explore the specific biomarkers of tumour growth and immune microenvironment.

Midnolin (MIDN) was discovered as a regulator of neurogenesis‐related genes [9]. Previous research had demonstrated that MIDN localised in the nucleus and cytoplasm [10]. MIDN was identified as a biomarker for Parkinson's disease by regulating parkin [11, 12]. Recently, MIDN had been reported to play a significant role in the process of cell differentiation by regulating immediate‐early genes [13, 14]. However, the function of MIDN in cancer was rarely reported. Kweon's work demonstrated that MIDN was an oncogene in liver cancer. They found that knocking down MIDN in liver cancer cells inhibited the growth through retinoic acid metabolism or lipid metabolism [15, 16]. Unfortunately, studies of MIDN in other cancers have not been reported. Excitedly, MIDN was required for normal lymphopoiesis and was essential for malignant B cell proliferation in gene‐edited mouse models [17]. These studies suggest that MIDN may be involved in immune regulation. Clinically, only approximately 20% of patients are sensitive to immunotherapy, and most patients do not benefit from the therapeutic modality [18, 19]. Based on the few studies of MIDN, we tentatively speculate that it may play a role in tumour growth and immunity. However, a systematically comprehensive pan‐cancer analysis is urgent to explore the diagnosable biomarker of MIDN for clinical tumours.

Our work is based on the new function of MIDN in biology. We utilised various public databases to investigate the valuable biomarker of MIDN in cancers. A comprehensive analysis of MIDN affected gene expression, prognosis, immunological markers, and immune cell infiltration was performed. Moreover, we conducted in vitro and in vivo experiments to validate the regulatory function of MIDN on cell proliferation. It is suggested that MIDN can be used to predict the prognosis of different cancer types, and its differential expression in human cancers is associated with immune checkpoint genes, which provide evidence for the potential role of MIDN in tumour immunity. We comprehensively analysed the prognostic value of MIDN in a wide range of tumours and assessed its potential in tumour progression and therapy.

## 2 Materials and Methods

### 2.1 Expression Analysis of MIDN

The mRNA expression data of MIDN in human normal and tumour tissues were downloaded from the Genotype‐Tissue Expression Project (GTEx) (https://gtexportal.org/home/) and The Cancer Genome Atlas (TCGA) database. For statistical analysis and graphical presentation of the normalised gene expression data of MIDN in cancer and normal tissues from TCGA and GTEx databases, the R package easyTCGA was used. Cancer type abbreviations used in this article: adrenocortical cancer (ACC), bladder cancer (BLCA), breast cancer (BRCA), cervical cancer (CESC), bile duct cancer (CHOL), colon cancer (COAD), large b‐cell lymphoma (DLBC), oesophageal cancer (ESCA), glioblastoma (GBM), head and neck squamous cell carcinoma (HNSC), kidney chromophobe (KICH), kidney clear cell carcinoma (KIRC), kidney papillary cell carcinoma (KIRP), acute myeloid leukaemia (AML), lower grade glioma (LGG), liver cancer (LIHC), lung adenocarcinoma (LUAD), lung squamous cell carcinoma (LUSC), mesothelioma (MESO), ovarian cancer (OV), pancreatic cancer (PAAD), pheochromocytoma and paraganglioma (PCPG), prostate cancer (PRAD), rectal cancer (READ), sarcoma (SARC), melanoma (SKCM), stomach cancer (STAD), testicular cancer (TGCT), thyroid cancer (THCA), thymoma (THYM), endometrioid cancer (UCEC), uterine carcinosarcoma (UCS), and ocular melanomas (UVM).

### 2.2 DNA Methylation Analysis

The UALCAN (http://ualcan.path.uab.edu/index.html) helped to evaluate the methylation of the MIDN promoter between tumour and paired normal tissues across cancers [20].

### 2.3 Protein Expression Analysis of MIDN

Histochemical staining images depicting MIDN in normal and tumour tissues of BRCA, CESC, COAD, GBM, HNSC, KICH, LIHC, LUAD, MESO, OV, PAAD, PRAD, STAD, TGCT, and THCA were obtained from the Human Protein Atlas database [21].

### 2.4 Single‐Cell Expression Analysis of MIDN

The TISCH database (http://tisch.comp‐genomics.org/home/) provides single‐cell sequencing results in various cancers [22]. The expression data of MIDN mRNA in different cell types of 79 datasets were downloaded and presented partially in figures. In addition, Umap plots can distinguish in which cell populations MIDN is significantly enriched.

### 2.5 Survival Prognosis Analysis of MIDN

The Kaplan–Meier (KM) plotter (http://kmplot.com/analysis/) was used to evaluate patients' survival in cancers with the threshold of half [23, 24]. Statistical significance was set at *p* < 0.05.

### 2.6 Gene Set Enrichment Analysis (GSEA)

Based on the expression level of MIDN, patients in each cancer type were divided into high and low groups with a threshold of half; the differentially expressed genes were used for GSEA [25]. The cancer‐related hallmark gene set file (h.all.v2023.2.Hs.symbols.gmt) was used in this study. Normalised Enrichment Score (NES) and False Discovery Rate (FDR) were calculated using the R package GSEA. The analysed results were summarised and graphically presented in the bubble plot by ggplot2.

### 2.7 Immune Cell Infiltration Analysis

With the help of the ESTIMATE database (https://bioinformatics.mdanderson.org/estimate/), immune score data were analysed in cancers [26]. To analyse the influence of MIDN expression and mutant status on immune cell infiltration in 33 cancer types, the SangerBox web application (http://www.sangerbox.com/) and TIMER 2.0 (http://timer.cistrome.org/) was utilised [27, 28]. The correlation between MIDN expression and the infiltration degree of 22 immune cell types, including B cells naive, B cells memory, plasma cells, T cells CD8, T cells CD4 naive, T cells CD4 memory resting, T cells CD4 memory activated, T cells follicular helper, T cells regulatory Tregs, T cells gamma delta, NK cells resting, NK cells activated, monocytes, macrophages M0, macrophages M1, macrophages M2, dendritic cells resting, dendritic cells activated, mast cells resting, mast cells activated, eosinophils, and neutrophils, was examined. Three progenitor types were explored using spearman correlation analysis.

### 2.8 Predictive Analysis of Immunotherapy Response

The expression correlation between MIDN and 60 immune‐related regulators in cancers was analysed. Moreover, the correlation between MIDN expression and tumour mutation burden (TMB) or microsatellite instability (MSI) was analysed using the SangerBox web application.

### 2.9 Cell Culture and Transfection

HEK293 and Huh‐7 cell lines were purchased from the American Type Culture Collection (ATCC). Huh‐7 was maintained in a Dulbecco's Modified Eagle Medium (DMEM) with 10% fetal bovine serum (FBS) and incubated at 37°C and 5% CO_2_. The sequences for knocking down MIDN were obtained from the GPP Web Portal (#1 GATGTGAACATCACGTGTTAT, #2 CAGAAGTCAACCCTGACATCA), and the shRNA sequences were subcloned into the pLenti‐U6‐puro‐MIDN plasmid. Transfection was carried out for 48 h. Huh‐7 cells were infected with MIDN/control shRNA viruses for 24 h in the presence of polybrene. Stable Huh‐7‐MIDN‐sh1/sh2 cells were generated via puromycin selection.

### 2.10 MTT Analysis

Huh‐7‐vector/MIDN‐sh1/MIDN‐sh2 cells were plated into 96‐well plates with 3000 cells/well. The detection method is referred to in the previous study. The result was applied every day to evaluate cell viability and finish the assay in 6 days [29].

### 2.11 Colony Formation

According to the previous method, MIDN model cells were prepared and seeded into 12‐well plates with 1000 cells/well. The plates were cultured for 7–10 days, and then the colonies were fixed and stained.

### 2.12 Western Blot

The Western blot method is similar to the previous work [30]. Proteins were separated by gel electrophoresis and were transferred to PVDF membranes. After blocking with 5% nonfat milk at room temperature for 1 h, membranes were probed with antibody solutions overnight at 4°C. Then, the transfer membrane was incubated with secondary antibody for 1 h. The protein band images were detected using an ECL kit. MIDN (18939‐1‐AP), β‐catenin (51067‐2‐AP), and GAPDH (60004‐1‐Ig) antibodies were purchased from Proteintech Group.

### 2.13 Subcutaneous Xenograft Model

In vivo experiments were conducted in accordance with the protocols approved by the Ethical Committee for Animal Experimentation of the Southern Medical University. SPF‐grade female nude mice were obtained from Guangdong Medical Laboratory Animal Center. The experimental approach is similar to the previous study [30]. Tumour volume was calculated using the formula: volume = (long diameter) × (short diameter) × (short diameter)/2. The animal experiment finished in 24 days before the long diameter reached 15 mm, and the tumour parameters were determined.

### 2.14 Statistical Analysis

Student's *t*‐test and one‐way ANOVA test were used for all statistical analyses. All the graphs with error bars or statistical significance in this study were performed by using the SPSS 20.0 (IBM, Chicago, IL, USA) software package. The correlation between MIDN and TMB/MSI genes was assessed by Spearman's correlation method. The correlation of MIDN and immune checkpoint genes was analysed by the Pearson correlation test. All results used at least three replicates. *p* values < 0.05 were considered significant.

## 3 Results

### 3.1 Expression of MIDN in Human Cancers

**FIGURE 1:**
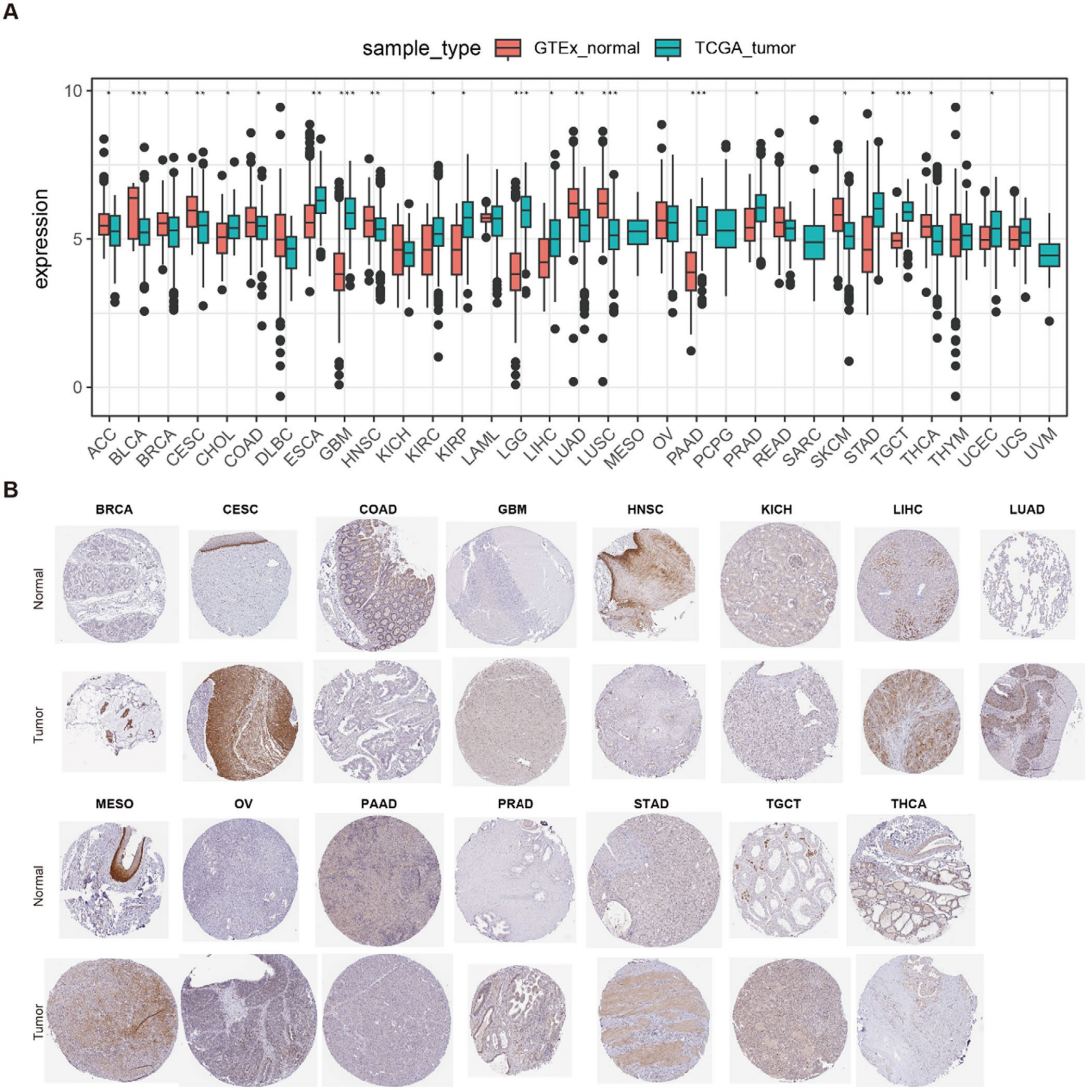
mRNA expression levels of *MIDN* in human cancers. (A) Boxplots showing the mRNA expression levels of MIDN in normal and cancer tissues using data from the TCGA database. Tumour tissues are represented by orange boxes, and normal tissues are represented by blue boxes. The symbols *, **, and *** indicate *p* < 0.05, *p* < 0.01, *p* < 0.001. (B) Representative images of immunohistochemical staining of MIDN in 15 types of normal and tumour tissues. IHC staining images were collected from the Human Protein Altas.

As the available numbers of normal tissues were too small compared to those of tumours, we combined the TCGA and GTEx databases to test the different expression pattern of MIDN in normal and tumour tissues. As shown in Figure 1A, mRNA levels of MIDN expression were downregulated in ACC, BLCA, CESC, COAD, LIHC, LUAD, LUSC, SKCM, and THCA. In contrast, it was upregulated in ESCA, GBM, KIRC, KIRP, LGG, PAAD, STAD, TGCT, and UCEC.

DNA methylation is the major epigenetic regulator before gene expression, especially the methylation of the promoter affects its function. According to the UALCAN database, we found a link between MIDN expression and the methylation level of the promoter with a variety of malignancies in different cancers. The promoter methylation results showed that tumour tissues from HNSC, KIRP, LIHC, LUAD, PAAD, and PRAD were lower than normal tissues (Figure S1).

Moreover, the protein levels of MIDN in BRCA, CESC, GBM, LIHC, LUAD, MESO, OV, PRAD, and TGCT cancers were higher than normal. In KICH and PAAD tumours, MIDN expression was detected to be lower than that in normal tissues (Figure 1B). Based on the mRNA and protein expression characteristics of MIDN, we suspected that the function of MIDN might be tissue specific. Their findings might contribute to the understanding of the development of cancer and provide therapeutic targets.

### 3.2 Prognostic Role of MIDN in Human Cancers

**FIGURE 2:**
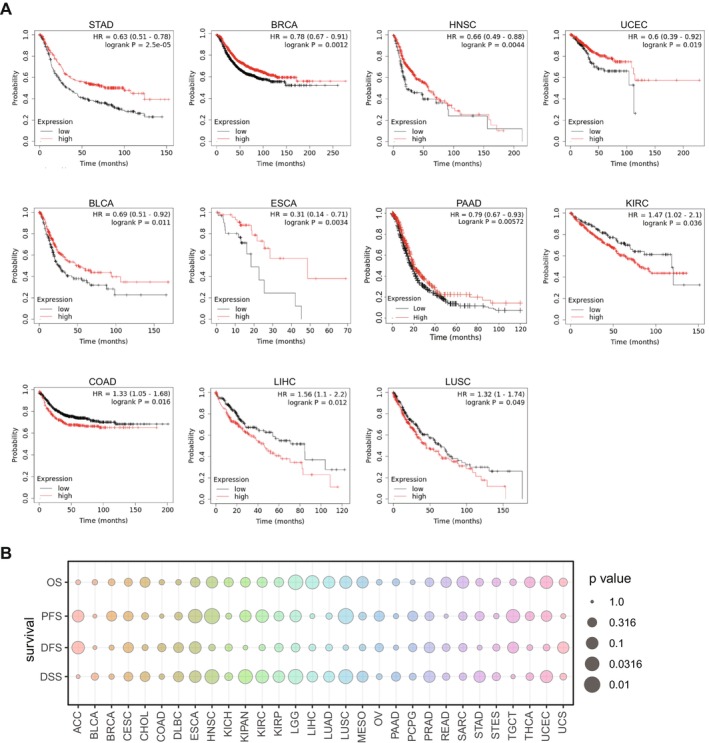
Prognostic role of MIDN in human cancers. (A) Representative survival curves of prognostic analysis comparing MIDN‐high and MIDN‐low patients in STAD, BRCA, HNSC, BLCA, ESCA, PAAD, COAD, LICH, LUSC, UCEC, and KIRC. (B) Heatmap showing the correlation between MIDN expression levels and four curated survival outcomes, including overall survival (OS), disease‐specific survival (DSS), disease‐free survival (DFS), and progression‐free survival (PFS). Survival analysis was performed using a log‐rank (KM) test and univariate Cox regression, based on curated survival data from the TCGA database.

The relationship between mRNA expression level of MIDN and prognosis in human cancers was analysed using Kaplan–Meier plotter. High expression of MIDN means better survival outcomes in STAD, BRCA, HNSC, BLCA, ESCA, and PAAD (*p* < 0.05). Besides, high expression of MIDN was linked to worse survival outcomes in COAD, LIHC, and LUSC (*p* < 0.05) (Figure 2A). Additionally, we analysed the overall survival (OS), progression‐free survival (PFS), disease‐free survival (DFS), and disease‐specific survival (DSS) for 30 cancers through the UCSC database. The expression of MIDN was a potential marker for prognosis in HNSC, LGG, LUSC, and LIHC (Figure 2B). Interestingly, the different prognostic roles of MIDN may be related to the organ microenvironment, and there is a need to explore the different mechanisms of MIDN's action in different tumours for clinical therapy.

### 3.3 GSEA Analysis of Potential Functions of MIDN in Human Cancers

To investigate the function of MIDN in cancers, we used GSEA to analyse MIDN‐related signalling pathway changes in multiple cancers. Analysis results indicated that stress response‐related pathways were significantly enriched, including unfolded protein response, TNFα signalling, and TGFβ signalling (Figure S2). These findings suggest that MIDN is a pivotal regulator in cancer progression [31].

Moreover, MIDN also contributes to cell proliferation in most cancers. Signalling pathways including MYC, mTORC1, Mitotic spindle, G2M, and E2F pathways are enriched in most cancers (Figure S2). Most of these pathways function in the nucleus. Similarly, MIDN has been identified as a gatekeeper of independent proteasome degradation in the nucleus [13]. The correlation may suggest that MIDN is important for cell proliferation, and it could function through controlling the stability of histone proteins or transcription factors. High expression of MIDN was positively correlated with DNA repair, p53 pathway, UV response, and mitotic spindle in many cancers. This correlation suggests that MIDN might function as a regulator of gene expression in the nucleus.

Finally, the immune‐related pathways were significantly regulated in most cancer patients with high expression of MIDN, including BLCA, COAD, ESCA, KICH, KIRP, LAML, LIHC, LUAD, LUSC, PAAD, PCPG, PRAD, STAD, THCA, and UCEC. Taken together, these findings indicated that elevated levels of MIDN might be a contributor to cell proliferation, epigenomic regulation, and immunity in various cancers.

### 3.4 MIDN Is Involved in the Regulation of Tumour Immunity

**FIGURE 3:**
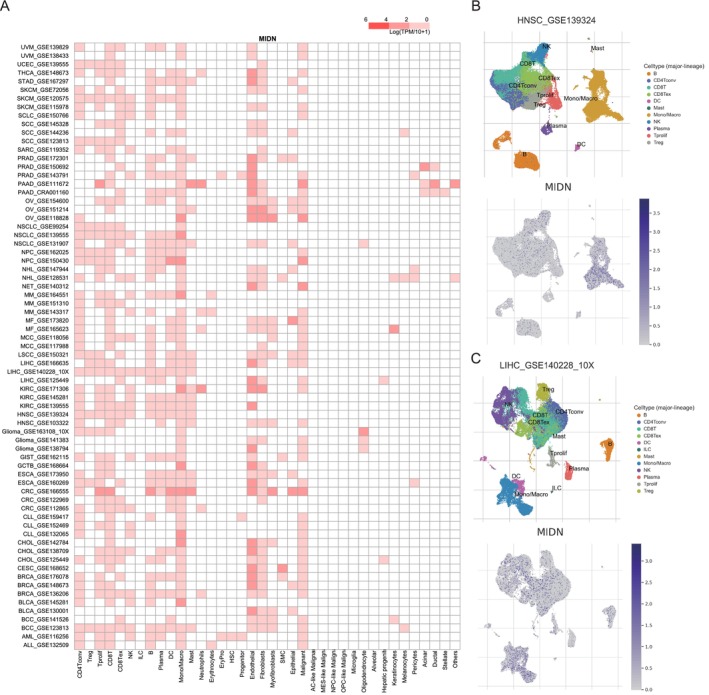
Single‐cell expression levels of MIDN in multiple cancer tissues. (A) Cluster heatmaps showing the mRNA levels of MIDN in 33 cell types of tumour tissues. (B, C) Umap plots displaying the clustering of different cell types (upper panel) and MIDN expression levels (lower panel) in HNSC (B) and LIHC (C) tissues.

To explore the cell types expressing MIDN in tumour tissues, we analysed the single‐cell expression data of MIDN using 79 datasets from the TISCH database. The results showed that MIDN was expressed in a variety of immune cells (Figure 3A). Notably, the analysis showed that MIDN expression was particularly pronounced in CD4^+^T, CD8^+^T, proliferating T cells, and B cells in HNSC (GSE139324), with a high expression level detected in monocytes/macrophages (Figure 3B). Similarly, MIDN was particularly highly expressed in monocytes/macrophages (Mono/Macro) according to the liver cancer dataset (GSE140228) (Figure 3C). Furthermore, the expression of MIDN in most cancers was closely related to CD4^+^ and CD8^+^ T cells, suggesting its potential regulatory role in tumour immunity (Figure S3).

### 3.5 The Expression and Mutation of MIDN Affect Immune Cell Infiltration

**FIGURE 4:**
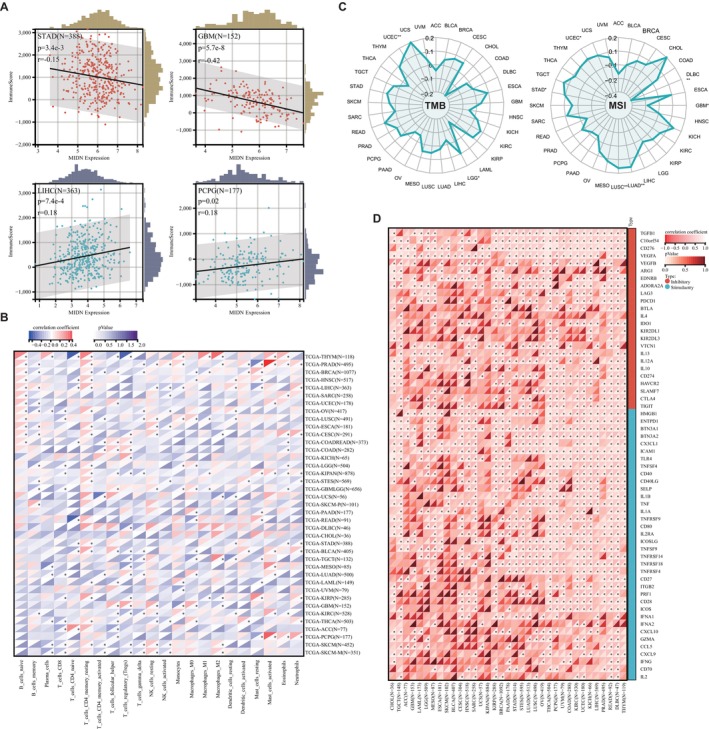
Correlation analysis between MIDN expression and immune cell infiltration. (A) The expression level of MIDN is negatively related to immune scores in STAD and GBM. The expression level of MIDN is positively related to immune scores in LIHC and PCPG. The immune scores were calculated through Estimate. (B) Cluster heatmaps display the correlation between MIDN expressions and the degree of infiltration by immune cells. *, *p* < 0.05. (C) Radar plots demonstrate the correlation between MIDN expression and microsatellite instability (MSI, up) and tumour mutation burden (TMB, down) of multiple tumour types. (D) Cluster heatmaps show the correlation analysis of the expression levels between MIDN and 60 immune genes in 33 cancer types.

To explore the relationship of MIDN and immunity in cancers, we divided patients into MIDN‐high and MIDN‐low expressing groups and then analysed the immune score in those groups by using the ESTIMATE database. The immune scores for the high MIDN‐expression cancers (including GBM, LUAD, BRCA, TGCT, THYM, SKCM, KIRP, SARC, LUSC, ACC, ESCA, and STAD) were significantly lower than those with low MIDN expression. Furthermore, MIDN was positively correlated with immune scores in LIHC, LAML, DLBC, and PCPG patients (Figure 4A and Figure S4). Our data were consistent with former GSEA analysis (Figure S2), underlining that MIDN may affect immunity in cancers.

To find the immune cell regulated by MIDN in human cancers, Pearson correlation analyses were performed utilising data from the CIBERSORT database. MIDN expression was positively associated with the infiltration of CD4^+^ memory resting T cells, Tregs, and macrophage cells, but negatively associated with memory B cells, CD8^+^ T cells, and gamma delta T cells (Figure 4B). Those results indicated that MIDN expression is beneficial to the immune microenvironment (TME). In fact, the relationship between MIDN and immunity varies in tissues.

As immune‐related regulators can be promising targets for cancer immunotherapy, we found the association of MIDN with 60 immune checkpoint‐associated genes in cancers. MIDN was positively correlated with most immunological checkpoints genes in major cancers. Notably, MIDN was negatively correlated with the majority of immunological checkpoints in CHOL, TGCT, ACC, GBM, and LAML (Figure 4D). Tumour mutation burden (TMB) and microsatellite instability (MSI) are defined as promising immunotherapy prediction biomarkers [32]. We discovered that MIDN was positively associated with TMB in LGG and UCEC. The significant association with MSI was detected in LUAD, LUSC, STAD, and UCEC and negatively correlated in DLBC and GBM (Figure 4C). Based on these results, we speculated that MIDN may have the potential to predict the response to immunotherapy in the corresponding cancers.

**FIGURE 5:**
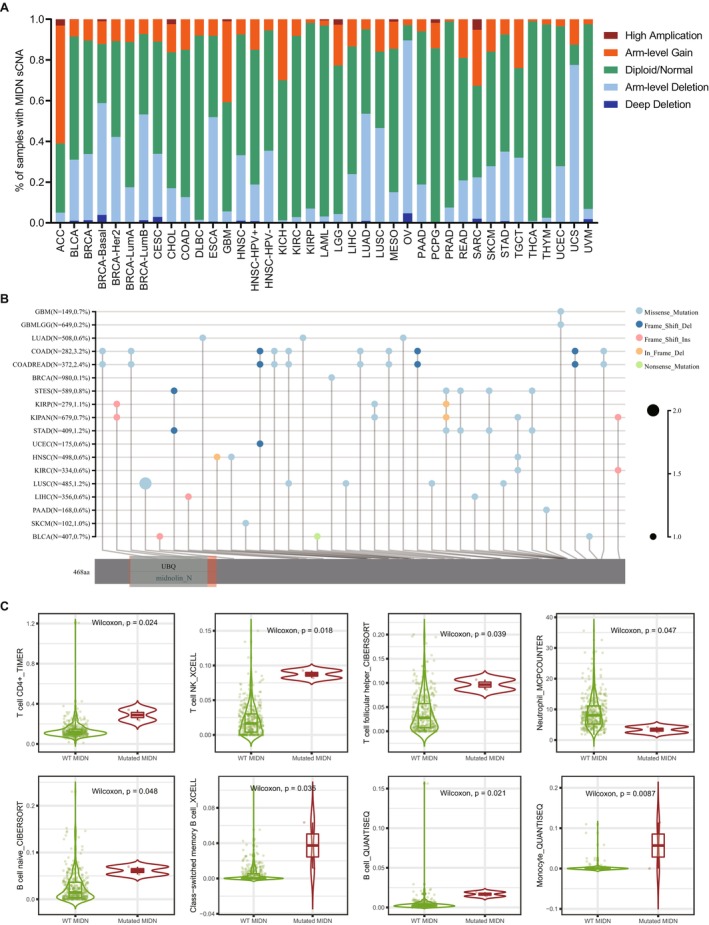
Mutation of MIDN affected immune cell infiltration in LIHC. (A) The distribution of MIDN's sCNA state in cancers, including “deep deletion”, “arm‐level deletion”, “diploid/normal”, “arm‐level gain”, and “high amplification”. (B) The mutation landscape of MIDN in 18 cancers. (C) The immune infiltration distribution between different sCNA statuses of MIDN in LIHC.

It is common to detect somatic copy number alterations (sCNA) in human cancers [33]. The role of sCNA in tumour immunity is multifaceted, involving immune evasion of tumour cells, regulation of immune checkpoints, infiltration of immune cells, and impacts on genomic stability. These factors collectively determine the response of tumours to immune therapy and the prognosis of patients. We analysed the sCNA of MIDN in various tumours and found amplification in tumours such as ACC, GBM, and SARC. However, genomic deletion was found in OV, UCS, ESCA, LUAD, and LUSC (Figure 5A). We categorised based on the amplification and deletion of MIDN and used various models to analyse the relationship between these mutations and immune cell infiltration. Our results showed that myeloid dendritic cells, CD8^+^ T cells, and macrophages were associated with the amplification and deletion of MIDN in BRCA, LUSC, and STAD (Figure S5). Comparing the mutation sites of the MIDN gene in 16 cancers, we found that the mutation sites of MIDN varied among different tumours (Figure 5B). These results suggested that the characteristics of MIDN in different tissues may affect its function. The mutation of MIDN was closely associated with the infiltration of immune cells. The analysis results from the TIMER 2.0 database indicated that the mutation of MIDN could promote infiltration of CD4^+^ T cells, Neutrophil cells, and monocyte in LIHC (Figure 5C). A notable correlation between immune cell infiltration and MIDN mutations could also be observed in other tumours (Figure S5).

### 3.6 Function of MIDN Relays on β‐Catenin and Its Association With MMP9 Suppresses Immune Cell Infiltration in LIHC

**FIGURE 6:**
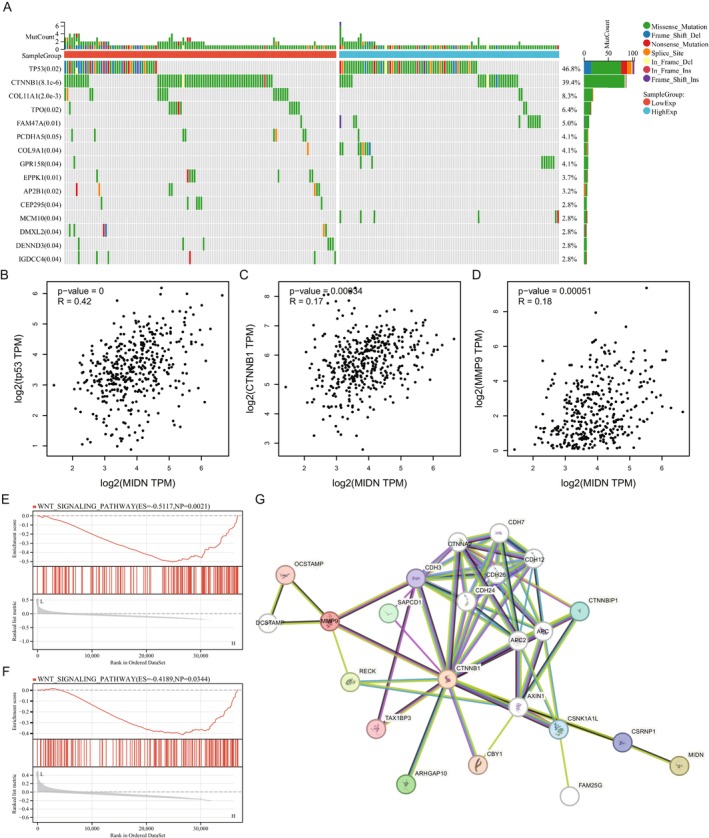
MIDN mutation in LIHC and function through CTNNB1. (A) Mutation landscape of MIDN in LIHC. The data were divided into two groups based on MIDN expression. (B–D) Correlation of MIDN and TP53, CTNNB1, and MMP9 expression in liver cancer. (E, F) GSEA analysis result indicated MIDN and MMP9 were significantly enriched in the Wnt signalling pathway, and CTNNB1 (β‐catenin) is a key role in this pathway. (G) STRING analysis result indicated the interaction among MIDN, CTNNB1, and MMP9.

Compared with other cancers, we found that the transcriptional expression of MIDN in liver cancer is consistent with the protein level, and its transcriptional level is significantly correlated with prognosis and immune cell infiltration. To further explore the function of MIDN in liver cancer, we used the TCGA‐LIHC dataset to identify mutations of key proteins that are closely related. We divided patients into high‐ and low‐MIDN expression groups; the mutation landscape showed that TP53 and CTNNB1 ranked first after analysis by Sangerbox (Figure 6A). Moreover, the expression of MIDN was significant with TP53 and CTNNB1 (Figure 6B,C). Somatic CTNNB1 mutations are present in about 27% of HCC patients [34]. Recently, Cai's work reported that CTNNB1 activates MMP9 to induce a suppressive tumour immune microenvironment [35]. Then, we explored the association of MIDN and MMP9 in mRNA expression level; the result demonstrated that the expression of MIDN was tightly related to the CTNNB1/MMP9 axis (Figure 6D). MIDN and MMP9 were significantly enriched in the Wnt signalling pathway after GSEA analysis in the TCGA‐LIHC dataset (Figure 6E,F). STRING analysis supported the regulative pathway from MIDN to MMP9 (Figure 6G). Together, these data supported that MIDN promotes progression in liver cancer and its function was associated with β‐Catenin.

### 3.7 Knockdown of MIDN Inhibits Liver Cancer Progression

**FIGURE 7:**
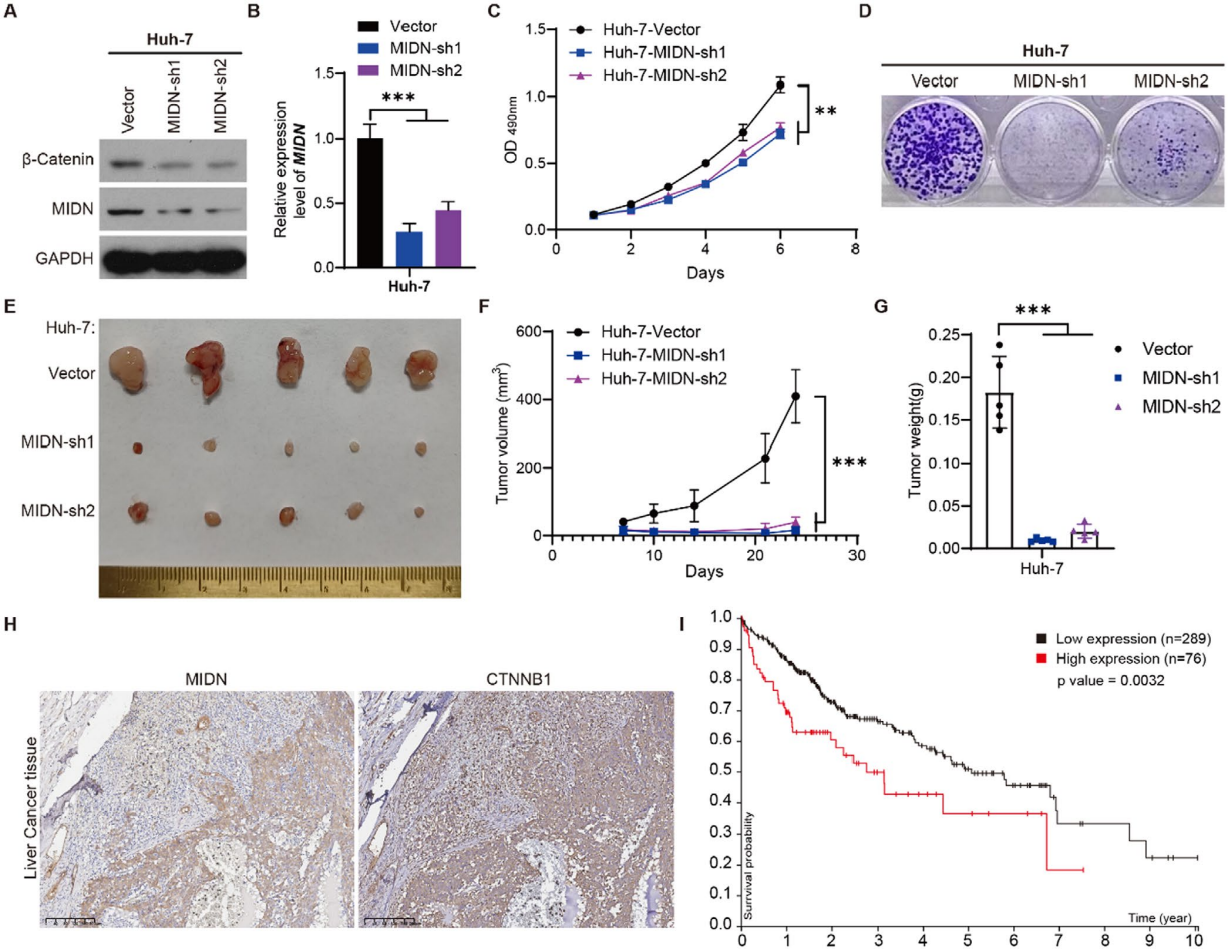
MIDN knockdown in LIHC inhibits the progression in vivo and in vitro. (A, B) Knockdown of MIDN remarkably reduces the protein and mRNA levels of MIDN in Huh‐7 cells. (C) MTT assay. (D) Colony formation. (E) Images of Huh‐7‐Vector and Huh‐7‐MIDN‐sh1/2 tumours derived from mice. *n* = 5 per group. (F) Growth curves of Huh‐7‐Vector and Huh‐7‐MIDN‐sh1/2 tumours. (G) Tumour weight of Huh‐7‐Vector and Huh‐7‐MIDN‐sh1/2 tumours. (H) IHC staining of MIDN and CTNNB1 in LIHC. (I) Prognostic analysis of CTNNB1 in liver cancer. Clinical data collected from the Human Protein Atlas database. The symbols ** and *** indicate *p* < 0.01, *p* < 0.001, respectively.

To investigate the cellular functions of MIDN, we employed a liver cancer cell line (Huh‐7) to investigate the role of MIDN. Previous TCGA analysis demonstrated that the expression of MIDN was higher in liver tumours (Figure 1A). The decrease in protein levels of CTNNB1 (β‐Catenin) in Huh‐7 cells was confirmed by Western blotting. We established stably MIDN knocked down Huh‐7 cells. The efficiency of MIDN knockdown was confirmed by Western blot and RT‐qPCR (Figure 7A,B). MTT and colony formation assays demonstrated a significant reduction in cell viability and proliferation in Huh‐7 cells with MIDN knocked down (Figure 7C,D). A subcutaneous xenograft model was used for confirming the function of MIDN in vivo. As shown in Figure 7E–G, the tumour sizes and growth rates of Huh‐7 tumours in nude mice were dramatically reduced after the expression of MIDN was knocked down, indicating that MIDN contributes to the progression of liver cancer. Of note, in vitro and in vivo experiment results were consistent with the previous analysis (Figure 1B and Figure 2A). In clinical liver tumours, IHC staining suggested that MIDN promotes the expression of CTNNB1 (Figure 7H). The high expression of CTNNB1 in liver cancer means poor prognosis (Figure 7I). The correlation of MIDN and CTNNB1 suggested that MIDN might be an important regulator in liver cancer.

## 4 Discussion

The current burden of cancer remains the greatest threat to global human health. Significant progress has been made in understanding the characteristics of cancer and preliminarily explaining the mechanisms of cancer progression in the historical process of fighting cancer [36]. While these partial substantial advances have been significant [37, 38], a deeper understanding of cancer biology will provide a theoretical basis and practical needs for cancer treatment in the future. Over the past decades, cancer has primarily been considered to be caused by genetic mutations, while also acknowledging the significant role of the tumour microenvironment [39]. The Cancer Genome Atlas (TCGA) database has integrated the genomic data of 33 common cancers, providing a foundation for investigations aimed at better understanding the pathogenesis of cancers. Fully utilising TCGA might help discover commonalities among cancers as well as tissue‐specific characteristics. Recently, Song's work demonstrated that dietary restriction was associated with tumour immunity in multiple cancers [40]. Their conclusions were also based on a model of gene expression associated with dietary restriction. Therefore, establishing models based on the TCGA database may facilitate an in‐depth understanding of tumours.

Previous studies have shown that MIDN responds to signals of low glucose and regulates the state of pancreatic beta cells [10]. Moreover, the high expression of MIDN in the liver, muscle, and brain has aroused the interest of researchers, and most of them linked it to the regulation of metabolic enzymes. Kweon's research demonstrated that MIDN corelates with poor prognosis in liver cancer [15]. Similarly, our data found that MIDN could be a negative biomarker for liver cancer, and it might promote the progression through β‐catenin. Several studies have demonstrated a strong correlation between the expression of MIDN and Parkinson's disease [11, 41]. 10.5% of patients with sporadic Parkinson's disease lacked one copy of MIDN in genome. Knocking out MIDN in cells increased the expression of parkin, which is considered as a major causative gene in Parkinson's disease [11]. Clinical evidence verified the genetic association of MIDN with PD development in a British population and in a Japanese population [42]. These findings suggest that the expression of MIDN contributes to pathological processes in humans and might be of significance for target therapies in the clinic.

In our current study, MIDN was found to be highly expressed in BRCA, CHOL, GBM, LIHC, PRAD, and STAD and lowly expressed in BLCA, COAD, HNSC, KICH, and LUSC. Combined with its prognostic biomarker, MIDN was significantly associated with progression in LIHC, BLCA, and HNSC. With the help of an MIDN knocked‐out mouse model, Kweon's [16] study illustrated that MIDN attenuated the severity of nonalcoholic fatty liver disease by reducing cholesterol and lipid metabolism. Kweon's [15] another study revealed that MIDN promotes liver cancer progression through retinoic and lipid metabolism. Taken together, abnormalities of MIDN in the liver are often associated with disease. Looking back at the results of big data analysis, it can be concluded that most digestive system neoplasms have a significant difference in the expression of MIDN. These data suggest that MIDN may be a biomarker for the dysfunction of tumour metabolism.

MIDN was identified as a key effector in the process of ubiquitination‐independent degradation [13]. MIDN promotes the degradation of nuclear proteins without ubiquitination modification [14]. Transcription factors can be rapidly degraded by MIDN, and it may be developed as a new tool for treatment [43]. Our data comprehensively analysed the relationship between MIDN expression and prognosis in multiple tumours. The tissue specificity of MIDN was comprehensively compared, and its positive and negative effects depend on the microenvironment. Immediate‐early genes are the targets of MIDN, and they are also very important in the regulation of cancer. Targeting immediate early gene expression and function remains an untapped area in cancer prevention research, and it could very well provide new resources in cancer treatment and new perspectives in directed cancer suppression [44].

Recently, Zhong's [17] study has shown that MIDN is essential for the differentiation of B cells. Their data tentatively suggest an important role for MIDN in B‐cell immunity. However, the immune microenvironment is particularly important for tumour growth, and various immune cells affect the progression of tumours. We analysed the immune infiltration in 33 cancers, and the mutation of MIDN is significantly associated with T cells or macrophages. In the GSEA analysis results, immune‐related pathways are enriched. The TMB and MSI correlation supports the regulatory role of MIDN in cancers. The tumour microenvironment (TME) is critical to the benefits of immunotherapy [45]. Our work explored the valuable functions of MIDN on T cells, NK cells, and macrophage infiltration, which underlined its promising value in immunotherapy [46]. In mechanism, we found that MIDN interacted with the CTNNB1/MMP9 axis, which was verified as an important pathway in suppressing the tumour immune microenvironment [35]. All data further prove that MIDN contributes to immunotherapy for various malignancies.

## 5 Conclusions

In conclusion, MIDN might represent a potential immunological and prognostic biomarker in cancers as indicated by our comprehensive analysis. MIDN appears to function in cell proliferation, while the protein expression levels were higher in BRCA, CESC, LIHC, LUAD, MESO, and STAD tissues. In view of tissue specificity, MIDN is seen to be a promoter in the development of PAAD, HNSC, and THCA. Notably, the mutation of MIDN in cancers was significantly associated with immune cell infiltration. In liver cancer, the MIDN/CTNNB1/MMP9 axis promotes progression through inducing a suppressive tumour immune microenvironment.

## Author Contributions

**Shaobo Huang:** conceptualization (equal), data curation (equal), formal analysis (equal), funding acquisition (equal), investigation (equal), writing – original draft (lead), writing – review and editing (equal). **Jinling Zhang:** data curation (equal), formal analysis (equal), investigation (supporting), methodology (equal), supervision (equal), validation (equal), writing – original draft (supporting). **Ting He:** formal analysis (supporting), methodology (supporting), software (supporting). **Jianping Zhou:** project administration (equal), resources (equal), writing – review and editing (equal). **Zhigang Liu:** conceptualization (equal), funding acquisition (equal), project administration (equal), resources (equal), writing – original draft (equal), writing – review and editing (equal).

## Conflicts of Interest

The authors declare no conflicts of interest.

## Supporting information


Figures S1–S5.

## Data Availability

Data available on request from the authors.
