# Supplementary material for: Midnolin Correlates With Anti‐Tumour Immunity and Promotes Liver Cancer Progression Through β‐Catenin
Source: J Cell Mol Med. 2025 Mar 20;29(6):e70472. doi: 10.1111/jcmm.70472 (PMC11924130; doi:10.1111/jcmm.70472)
Supplement: Supplementary file 1 — Figures S1–S5. [file JCMM-29-e70472-s001.docx]

**Supplementary Material**


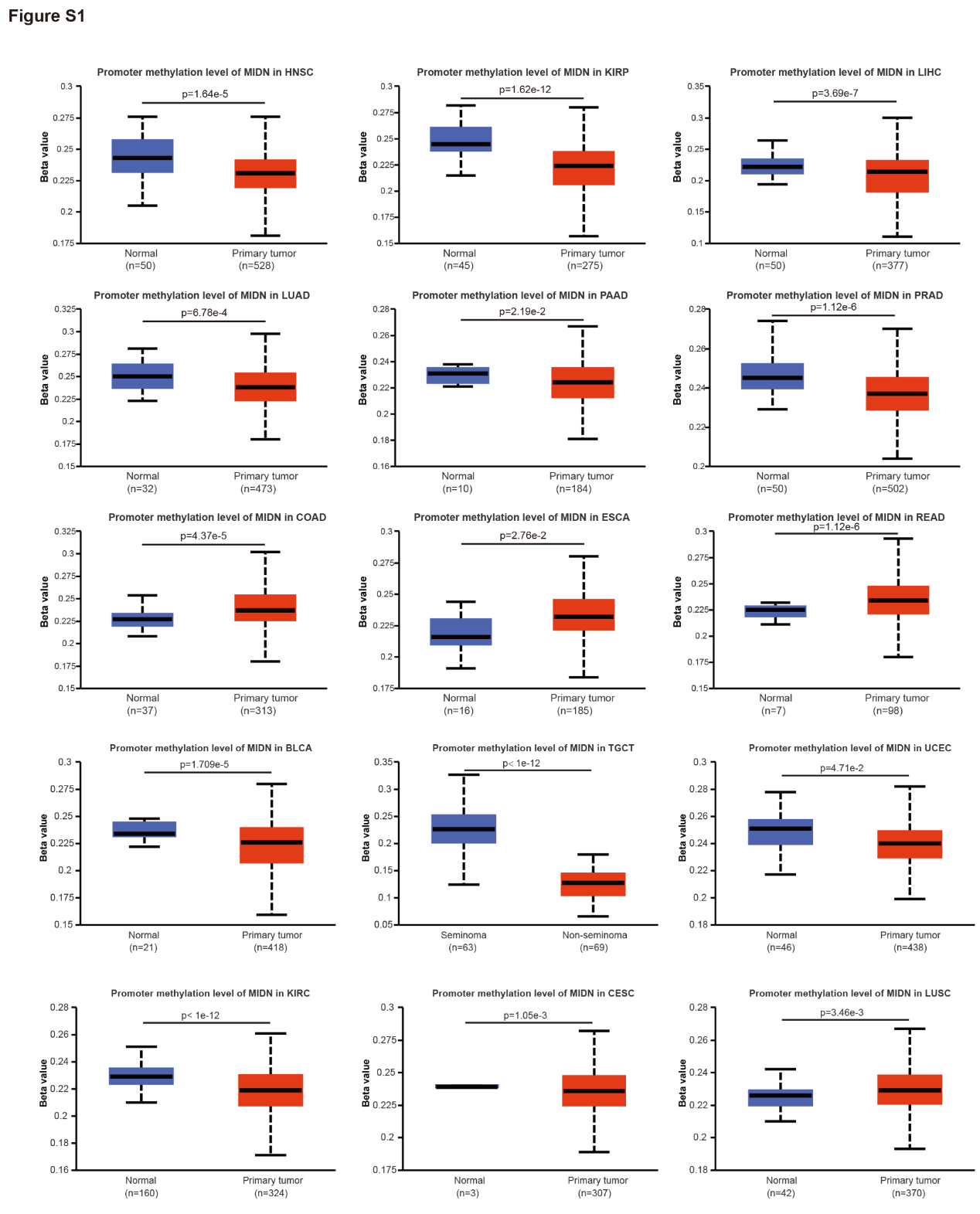


**Supplementary Figure S1. Methylation of MIDN promoter in normal and tumor tissues.**


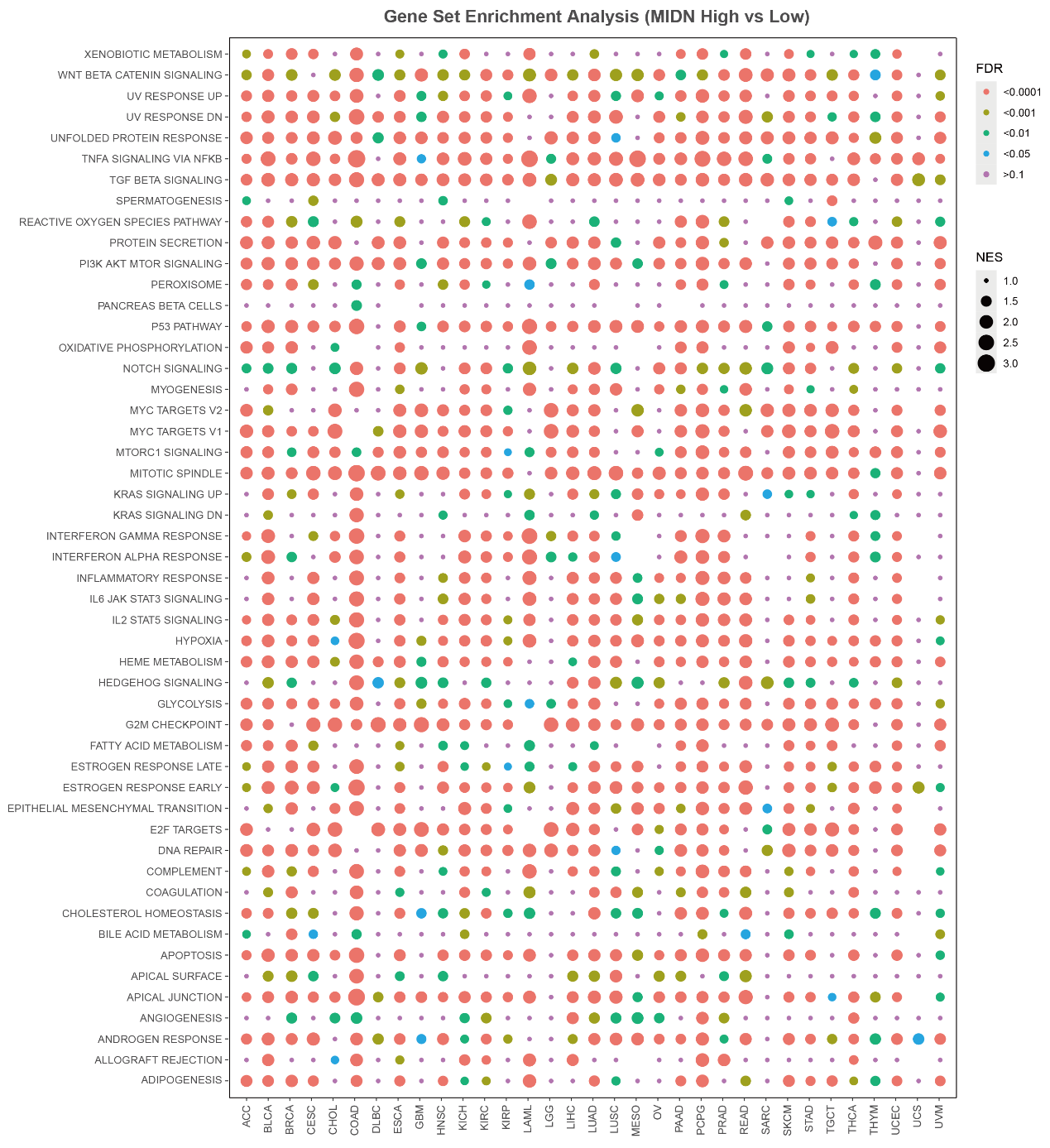


**Supplementary Figure S2. GSEA analysis of potential functions of MIDN in human cancers**. A bubble plot shows the results of GSEA between MIDN-high and -low tumor patients using hallmark gene signatures. The color codes from blue to red represents the magnitude of the p value, while the size of each circle represents the magnitude of the normalized enrichment scores (NES).


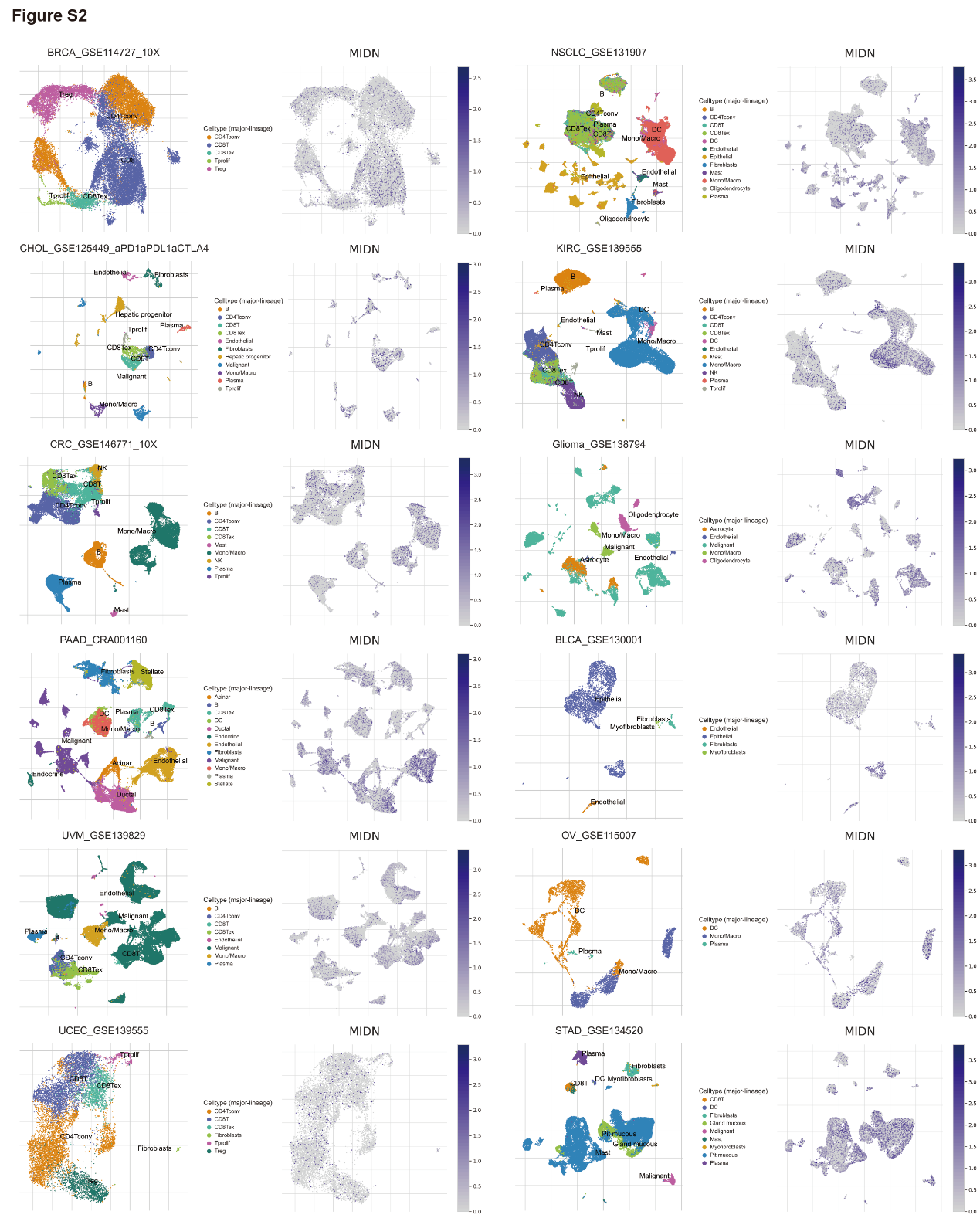


**Supplementary Figure S3. Single-cell expression levels of MIDN in multiple cancer tissues.** Umap plots displaying the clustering of different cell types (left panel) and MIDN expression level (right panel) in other tumor tissues. BRCA (GSE114727), NSCLC (GSE131907), CHOL (GSE125449), KIRC (GSE139555), CRC (GSE146771), GBM (GSE138794), PAAD (CRA001160), BLCA (GSE130001), UVM (GSE139829), OV (GSE115007), UCEC (GSE139555) and STAD (GSE134520).


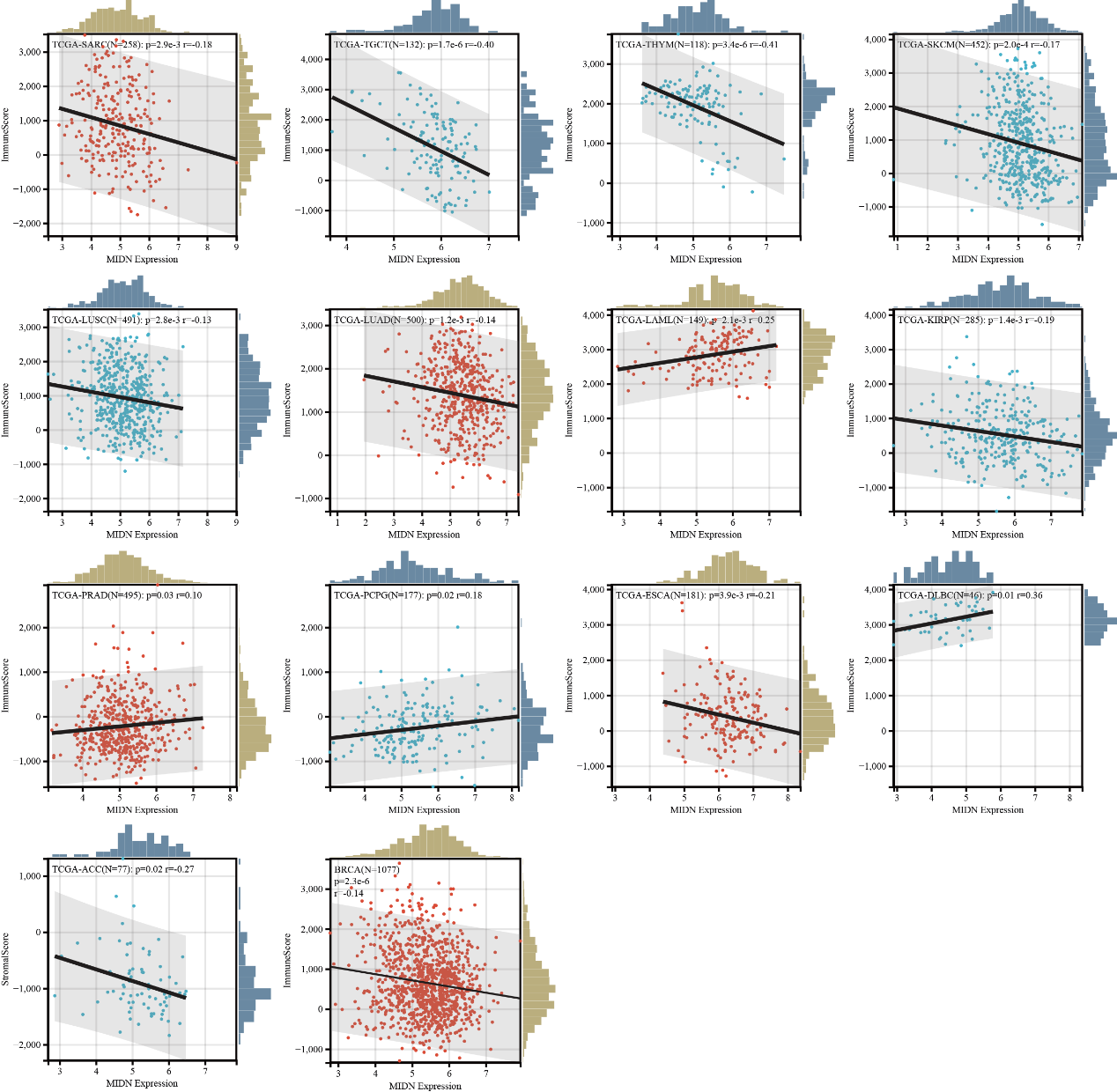


**Supplementary Figure S4. The comparison of immune scores between MIDN-high and MIDN-low patients through Estimate.** Sarcoma (SARC), testicular cancer (TGCT), thymoma (THYM), melanoma (SKCM), lung squamous cell carcinoma (LUSC), lung adenocarcinoma (LUAD), acute myeloid leukemia (LAML), kidney papillary cell carcinoma (KIRP), prostate cancer (PRAD), pheochromocytoma & paraganglioma (PCPG), esophageal cancer (ESCA), large b-cell lymphoma (DLBC), adrenocortical cancer (ACC), breast cancer (BRCA).


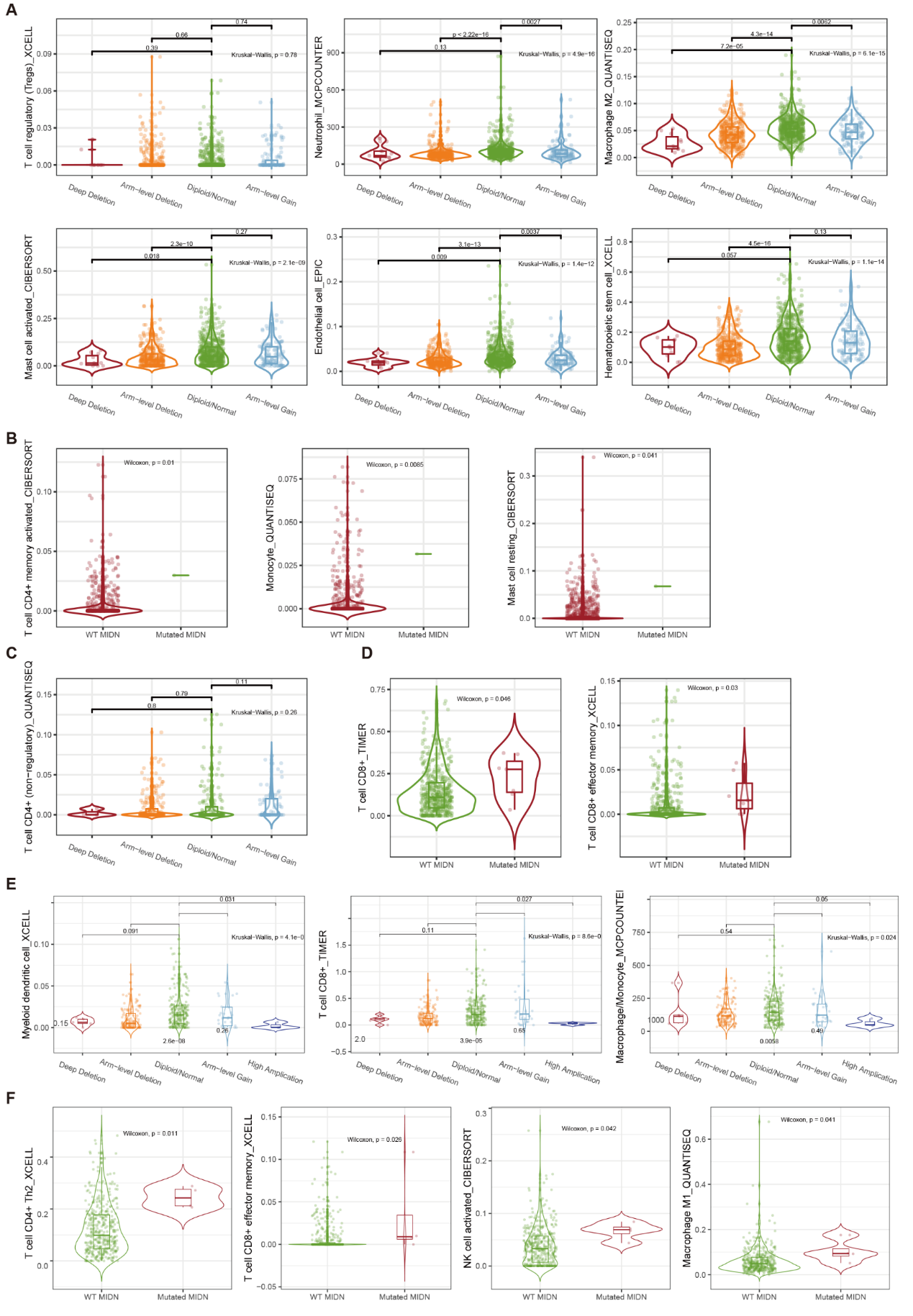


**Supplementary Figure S5. Mutation of MIDN affected immune cell infiltration in cancers.** (A) The amplification and deletion of MIDN was associated with Tregs cells, Neutrophil cells, and macrophages in BRCA. (B) The immune infiltration distribution between wilt and mutated MIDN in BRCA. (C) The amplification and deletion of MIDN was associated with CD4^+^ T cells in LUSC. (D) The immune infiltration distribution between wilt and mutated MIDN in LUSC. (E) The amplification and deletion of MIDN was associated with CD4^+^ T cells in STAD. (F) The immune infiltration distribution between wilt and mutated MIDN in STAD.
